# Remnant cholesterol is associated with cardiovascular mortality

**DOI:** 10.3389/fcvm.2022.984711

**Published:** 2022-09-20

**Authors:** Kerui Zhang, Xiangyun Qi, Fuyu Zhu, Quanbin Dong, Zhongshan Gou, Fang Wang, Li Xiao, Menghuan Li, Lianmin Chen, Yifeng Wang, Haifeng Zhang, Yanhui Sheng, Xiangqing Kong

**Affiliations:** ^1^Cardiovascular Research Center, The Affiliated Suzhou Hospital of Nanjing Medical University, Suzhou Municipal Hospital, Gusu School, Nanjing Medical University, Suzhou, China; ^2^Department of Cardiology, The First Affiliated Hospital of Nanjing Medical University, Nanjing Medical University, Nanjing, China; ^3^Department of Genetics, University Medical Center Groningen, University of Groningen, Groningen, Netherlands

**Keywords:** remnant cholesterol, Sampson formula, cardiovascular mortality, NHANES, correlational analysis

## Abstract

**Background:**

Genetic, observational, and clinical intervention studies indicate that circulating levels of remnant cholesterol (RC) are associated with cardiovascular diseases. However, the predictive value of RC for cardiovascular mortality in the general population remains unclear.

**Methods:**

Our study population comprised 19,650 adults in the United States from the National Health and Nutrition Examination Survey (NHANES) (1999–2014). RC was calculated from non-high-density lipoprotein cholesterol (non-HDL-C) minus low-density lipoprotein cholesterol (LDL-C) determined by the Sampson formula. Multivariate Cox regression, restricted cubic spline analysis, and subgroup analysis were applied to explore the relationship of RC with cardiovascular mortality.

**Results:**

The mean age of the study cohort was 46.4 ± 19.2 years, and 48.7% of participants were male. During a median follow-up of 93 months, 382 (1.9%) cardiovascular deaths occurred. In a fully adjusted Cox regression model, log RC was significantly associated with cardiovascular mortality [hazard ratio (HR) 2.82; 95% confidence interval (CI) 1.17–6.81]. The restricted cubic spline curve indicated that log RC had a linear association with cardiovascular mortality (p for non-linearity = 0.899). People with higher LDL-C (≥130 mg/dL), higher RC [≥25.7/23.7 mg/dL in males/females corresponding to the LDL-C clinical cutoff point (130 mg/dL)] and abnormal HDL-C (<40/50 mg/dL in males/females) levels had a higher risk of cardiovascular mortality (HR 2.18; 95% CI 1.13–4.21 in males and HR 2.19; 95% CI 1.24–3.88 in females) than the reference group (lower LDL-C, lower RC and normal HDL-C levels).

**Conclusions:**

Elevated RC levels were associated with cardiovascular mortality independent of traditional risk factors.

## Introduction

Abnormal lipid metabolism plays a key role in atherosclerosis, causing adverse cardiovascular events (1–4). A reduction in the level of low-density lipoprotein cholesterol (LDL-C) was thought to diminish the morbidity and mortality of cardiovascular events. However, it is apparent that there is a residual risk not attributable to LDL-C or other known risk factors (5).

Remnant cholesterol (RC), which is defined as the cholesterol present in triglyceride-rich remnant lipoproteins (TRLs) (6), has been demonstrated to be associated with atherosclerosis development and total mortality in several observational studies (7–9). About 1/3 of the cholesterol load carried by apolipoprotein B (apoB) containing lipoprotein particles is transported *via* remnant particles in non-fasting conditions. And, cardiovascular risk remains high in statin-treated individuals, even with low LDL-C levels (10, 11). These suggest that RC, rather than LDL-C, is an independent risk factor for cardiovascular diseases (CVDs).

However, there are significant hurdles in investigating and quantifying the contribution of RC to atherogenesis (12). The spectrum of TRLs in the circulation is heterogenous, and there is no definitive biomarker that permits the unequivocal quantitation of remnant levels. This is partly because TRLs in the circulation are modified by metabolic processes (13). Accordingly, it is reasonable to calculate RC by using other available lipid data. The calculation of LDL-C is an essential step. Recently, various methods have been used for determining LDL-C. However, direct methods of LDL-C measurement, such as the β-quantification procedure, require specialized equipment and a large volume of serum. In contrast to ultracentrifugation, other methods of isolating LDL-C use proprietary chemicals that lack standardization, adding time and expense to the process (14).

The National Health and Nutrition Examination Survey (NHANES) is a continuous program administered by the National Center for Health Statistics (NCHS), which is responsible for the nation's health and vital statistics. However, the National Death Index record linked to NHANES is updated only to December 31, 2015, and LDL-C was determined by the Friedewald equation instead of direct tests. By this method, the RC value is essentially one-fifth of the triglyceride (TG) value, and other components are not counted (15). Thus, estimating LDL-C accurately is imperative. Indirect methods for estimating LDL-C, such as the Martin equation, are recommended by guidelines (16). Unfortunately, LDL-C values derived from the Martin equation are inaccurate with TG values ≥400 mg/dL (17). In 2020, Sampson et al. developed a new formula for calculating LDL-C, the Sampson formula. Even when the TG level reached 800 mg/dL, the measurement results were almost consistent with direct methods and had superior accuracy to other indirect methods (18).

To our knowledge, the association of RC and long-term cardiovascular deaths has not yet been studied in the general population. Therefore, using clinical data from NHANES, we assessed the association of RC with cardiovascular mortality in the U.S. adult population.

## Methods

### Study design and population

**Figure 1:**
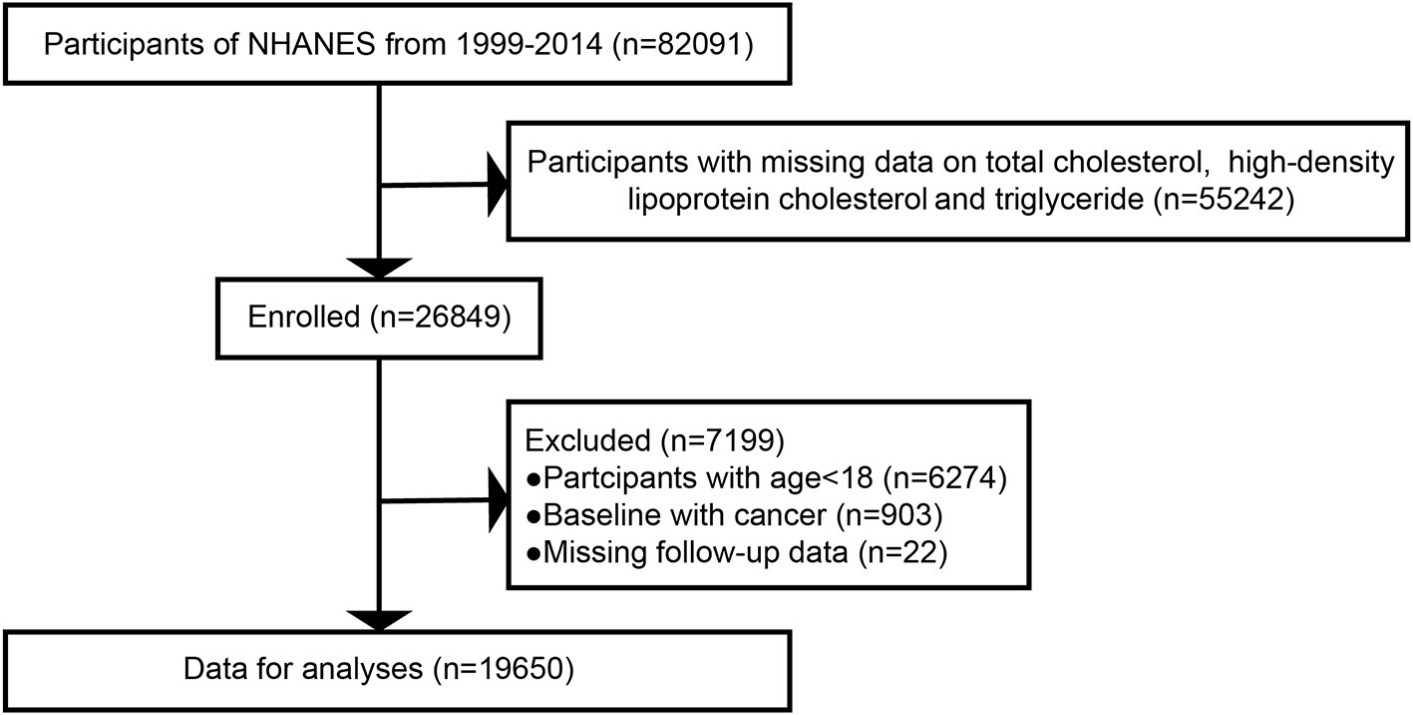
Study flow chart. NHANES, National Health and Nutrition Examination Survey.

Using a complex sampling design, the NHANES sample represents the non-institutionalized civilian population of the U.S. There were 82,091 participants in the survey cycles from 1999 to 2014. We included participants with available data on total cholesterol (TC), high-density lipoprotein cholesterol (HDL-C) and triglycerides (TGs) (*n* = 55,242). After the exclusion of participants aged <18 years (*n* = 6,274), those with cancer at baseline (*n* = 903) and those with missing follow-up data (*n* = 22), 19,650 individuals were enrolled for further analysis (Figure 1). All participants provided informed consent, and the program passed ethical review.

### Assessment of exposure

TC and TG were measured enzymatically, while HDL-C was quantified *via* the heparin-manganese precipitation method or direct immunoassay technique (19). LDL-C was estimated by the Sampson formula (18): LDL—C (mg/dL) = (TC/0.948) — (HDL—C/0.971) — [(TG/8.56) + (TG × non- HDL—C/ 2,140) — (TG × TG/16,100)] — 9.44.RC = non- HDL—C — LDL—C = TC — HDL—C — LDL—C (10, 11). All blood samples were collected using standardized procedures, and lipid concentrations were measured utilizing a Hitachi 704 Analyzer (20).

### Covariates

Demographic data, including age, sex and race, were collected by questionnaires. People who had smoked over 100 cigarettes during their lifetime were regarded as smokers (21). A history of present illness (e.g., heart failure, coronary artery disease and stroke) and medication treatment were obtained from self-reported personal interview data. Body mass index (BMI) was calculated using the following formula: weight (kg)/height squared (m^2^). Blood pressure (BP) was calculated by averaging three consecutive BP readings after the participants rested calmly for 5 min. Hypertension was defined as systolic BP ≥140 mmHg and/or diastolic BP ≥90 mmHg, a history of hypertension or use of antihypertensive drugs (22). Diabetes was defined as fasting blood glucose ≥7.0 mmol/L, hemoglobin A1c (HbA1c) ≥6.5% or a history of diabetes (23).

### Ascertainment of outcomes

The outcome of interest was cardiovascular mortality, defined as death caused by CVD or cerebrovascular disease. The death status and cause of death were identified by linking the National Death Index record to NHANES (reference for death registry). Follow-up lasted from the date of survey participation to cardiovascular mortality, the drop-out date, or December 31, 2015, whichever came first.

### Statistical analysis

Continuous and categorical variables were expressed as the mean ± standard deviation and numbers with percentages, respectively. Cox proportional hazards models were constructed to explore the association between the RC level (log_10_-transformed) and cardiovascular death. Model 1 was a crude model without adjustment for confounders. Age, sex, race, and use of medications were adjusted in model 2. Model 3 was additionally adjusted by several traditional cardiovascular risk factors, including SBP, coronary heart disease, stroke and smoking status. Considering that LDL-C and HDL-C are also important predictors of cardiovascular mortality, we constructed additional models. Models 4 and 5 were further adjusted by HDL-C and LDL-C, respectively. We incorporated both in model 6. Variables such as obesity (i.e., BMI) and diabetes, which are implicated in the causal pathway in rising RC levels, were not included in the models, nor were variables closely associated with the diagnosis of diabetes, including fasting blood glucose and HbA1c. For subgroup analysis, the results stratified by age, sex, diabetes, hypertension, and smoking status from the fully adjusted regression models were tested. Restricted cubic splines were used to explore the potential non-linear relationship of log RC with cardiovascular mortality in the total population and different subgroups. Multiple imputation was used to replace missing values. Statistical analyses were performed *via* R version 3.5.3. A *P*-value < 0.05 was regarded as statistically significant.

## Results

### Baseline characteristics

A total of 19,650 participants were ultimately included in this study (Table 1). The mean age was 46.4 ± 19.2 years, and 48.7% of participants were male. The median follow-up was 93 months, and 382 (1.9%) incident cardiovascular deaths occurred. Individuals with cardiovascular events were older, had higher SBP, HbA1c and fasting blood glucose levels, and accounted for a larger proportion of the diabetes, hypertension and antihypertensive and lipid-lowering drug use groups (*P* < 0.001) than those without cardiovascular events. In addition, they had higher TG and TC levels but lower HDL-C levels. Significantly, people who died of cardiovascular events had higher RC levels than other participants (*P* < 0.001).

**Table 1:** Baseline characteristics of the study population.

	**No cardiovascular events**	**Cardiovascular events**	***P*-value**	**Total**
Number	19268	382		19650
Age, years	46.0 ± 19.0	70.7 ± 12.6	<0.001	46.4 ± 19.2
Sex-male, *n* (%)	9,325 (48.4%)	243 (63.6%)	<0.001	9,568 (48.7%)
**Race**, ***n*** **(%)**			<0.001	
Mexican American	3,836 (19.9%)	62 (16.2%)		3,898 (19.8%)
Non-Hispanic white	8,562 (44.4%)	222 (58.1%)		8,784 (44.7%)
Non-Hispanic black	3,979 (20.7%)	74 (19.4%)		4,053 (20.6%)
Other races	2,891 (15.0%)	24 (6.3%)		2,915 (14.8%)
Smoking, *n* (%)	8,697 (45.1%)	234 (61.4%)	<0.001	8,931 (45.5%)
Coronary heart disease, *n* (%)	2,129 (11.0%)	81 (21.3%)	<0.001	2,210 (11.2%)
Stroke, *n* (%)	1,418 (7.4%)	39 (10.3%)	0.144	1,457 (7.4%)
Diabetes, *n* (%)	2,725 (14.1%)	143 (37.4%)	<0.001	2,868 (14.6%)
Hypertension, *n* (%)	7,026 (36.5%)	278 (72.8%)	<0.001	7,304 (37.2%)
BMI, kg/m^2^	28.5 ± 6.7	28.6 ± 6.2	0.33	28.5 ± 6.7
Systolic blood pressure, mmHg	122.2 ± 18.5	138.8 ± 24.3	<0.001	122.5 ± 18.7
Diastolic blood pressure, mmHg	69.6 ± 11.6	69.0 ± 13.7	0.448	69.6 ± 11.6
Blood glucose, mmol/L	5.84 ± 1.90	7.05 ± 3.22	<0.001	5.86 ± 1.94
Hemoglobin A1c, %	5.62 ± 1.02	6.27 ± 1.59	<0.001	5.63 ± 1.04
**TC**			0.024	
mmol/L	5.0 ± 1.1	5.2 ± 1.3		5.0 ± 1.1
mg/dL	194.5 ± 42.8	200.0 ± 48.4		194.6 ± 43.0
**HDL-C**			0.008	
mmol/L	1.4 ± 0.4	1.3 ± 0.4		1.4 ± 0.4
mg/dL	53.4 ± 15.8	51.6 ± 15.8		53.3 ± 15.8
**Non-HDL-C**			0.004	
mmol/L	3.7 ± 1.1	3.8 ± 1.3		3.7 ± 1.1
mg/dL	141.2 ± 42.6	148.4 ± 48.6		141.3 ± 42.8
**TG**			<0.001	
mmol/L	1.5 ± 1.3	1.9 ± 1.9		1.5 ± 1.3
mg/dL	135.2 ± 116.3	165.1 ± 165.2		135.7 ± 117.5
**LDL-C**			0.232	
mmol/L	3.0 ± 0.9	3.1 ± 1.0		3.0 ± 0.9
mg/dL	116.9 ± 36.2	119.2 ± 39.7		117.0 ± 36.3
**RC**			<0.001	
mmol/L	0.6 ± 0.5	0.8 ± 0.6		0.6 ± 0.5
mg/dL	24.2 ± 18.5	29.3 ± 23.8		24.3 ± 18.7
Antihypertensive medications, *n* (%)	4,932 (25.6%)	218 (57.1%)	<0.001	5,150 (26.2%)
Lipid-lowering medications, n (%)	6,676 (34.7%)	194 (50.8%)	<0.001	6,870 (35.0%)

*n*, number; TC, total cholesterol; HDL-C, high-density lipoprotein cholesterol; TG, triglycerides; LDL-C, low-density lipoprotein cholesterol; Values are mean ± standardized differences or *n* (%). To convert to SI units: TC, HDL-C, non- HDL-C, LDL-C and RC, multiply by 0.02586; to convert TG to SI units, multiply by 0.01129.

### Association of RC with cardiovascular mortality

**Figure 2:**
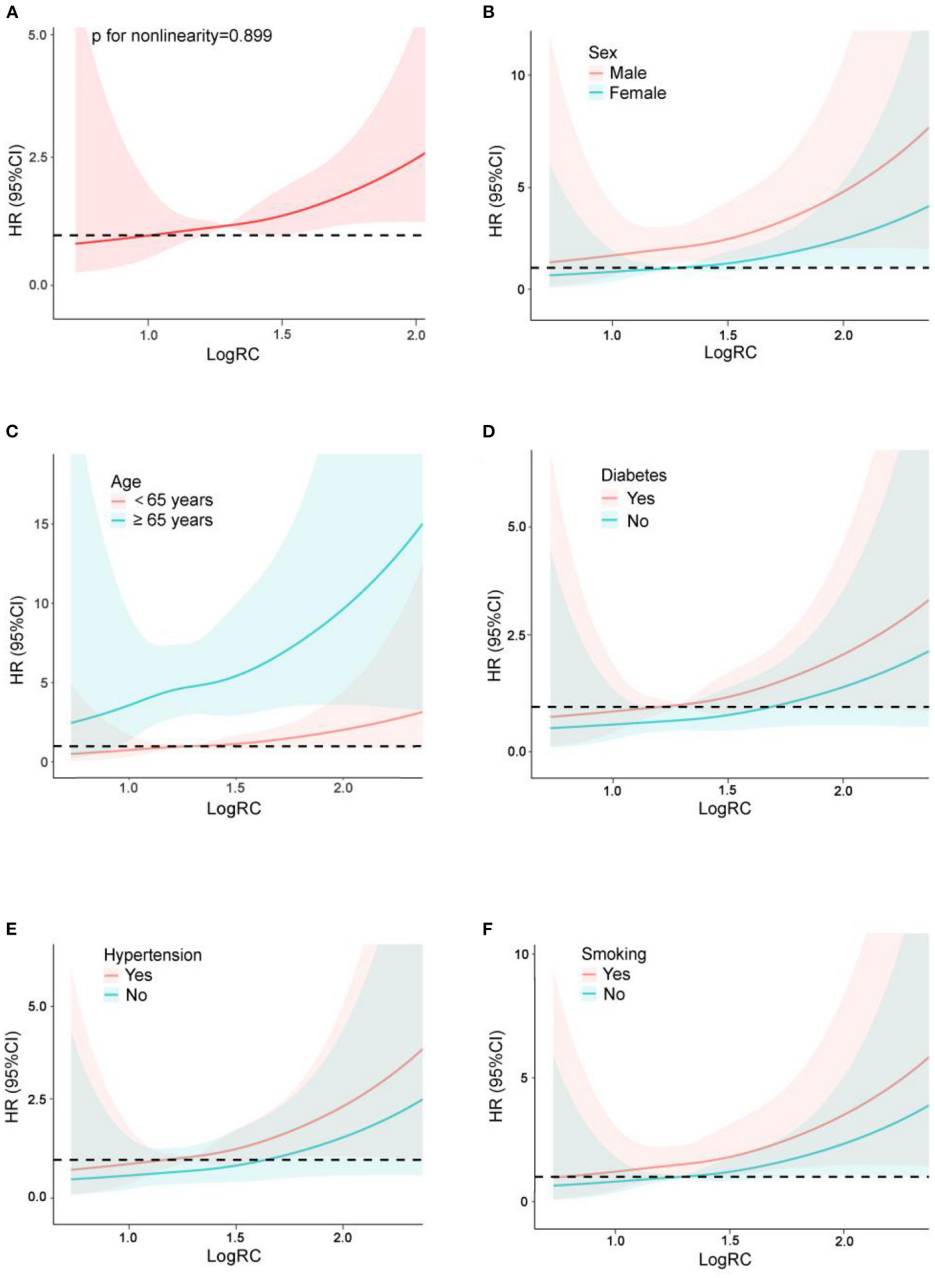
Restricted cubic spline plots of the association between log_10_-transformed RC and cardiovascular mortality in the general population **(A)** and subgroup analysis by sex **(B)**, age **(C)**, diabetes **(D)**, hypertension **(E)** and smoking **(F)**. Analysis was adjusted for age + sex + race + antihypertensive medications + lipid-lowering medications use + systolic blood pressure + coronary heart disease + stroke + smoking. HR, hazard ratio; CI, confidence interval.

After full adjustment for confounders as well as several traditional cardiovascular risk factors, our continuous analysis revealed a significant association between log RC levels (HR 2.82; 95% CI 1.17–6.81) and cardiovascular mortality. By comparison, the association of HDL-C and cardiovascular mortality was attenuated after adjustment for the same factors along with LDL-C (Table 2). Notably, RC was still associated with incident cardiovascular deaths despite adjustment for the risk factors directly related to the Sampson equation, including both HDL-C and LDL-C (all *p* < 0.05), although the significance was attenuated after additional adjustment (Table 3). The restricted cubic spline curve showed that log RC was linearly associated with cardiovascular mortality in the general population (p for non-linearity = 0.899) (Figure 2A).

**Table 2:** Cox models for cardiovascular mortality for logRC, RC, HDL-C, LDL-C (continuous variables) in the pooled cohort.

	**Model 1** **HR (95% CI)**	***P*-value**	**Model 2** **HR (95% CI)**	***P*-value**	**Model 3** **HR (95% CI)**	***P*-value**
LogRC	3.19 (2.17, 4.70)	<0.001	2.74 (1.62, 4.61)	<0.001	2.82 (1.17, 6.81)	0.021
**RC**		<0.001		<0.001		<0.001
mg/dL	1.01 (1.00, 1.01)		1.01 (1.00, 1.01)		1.01 (1.00, 1.01)	
mmol/L	1.30 (1.16, 1.47)		1.36 (1.16, 1.59)		1.39 (1.11, 1.72)	
**HDL-C**		0.064		0.007		0.537
mg/dL	0.99 (0.99, 1.00)		0.99 (0.98, 1.00)		1.00 (0.98, 1.01)	
mmol/L	0.78 (0.60, 1.01)		0.64 (0.48, 0.88)		0.85 (0.50, 1.44)	
**LDL-C**		0.707		0.876		0.285
mg/dL	1.00 (1.00, 1.00)		1.00 (1.00, 1.00)		1.00 (1.00, 1.01)	
mmol/L	1.02 (0.92, 1.14)		0.99 (0.88, 1.12)		1.12 (0.91, 1.38)	

HR, hazard ratio; CI, confidence interval. Model 1: non-adjusted. Model 2: adjusted for age + sex + race + antihypertensive medications + lipid-lowering medications use. Model 3: Model 2 + systolic blood pressure + coronary heart disease + stroke + smoking.

**Table 3:** Cox models for cardiovascular mortality for logRC, RC (continuous variables) after additional adjustment in the pooled cohort.

	**Model 4** **HR (95% CI)**	***P*-value**	**Model 5** **HR (95% CI)**	***P*-value**	**Model 6** **HR (95% CI)**	***P*-value**
LogRC	3.10 (1.16, 8.27)	0.024	2.72 (1.11, 6.65)	0.028	2.97 (1.10, 8.04)	0.032
**RC**		0.005		0.003		0.005
mg/dL	1.01 (1.00, 1.01)		1.01 (1.00, 1.01)		1.01 (1.00, 1.01)	
mmol/L	1.39 (1.10, 1.74)		1.40 (1.12, 1.75)		1.40 (1.11, 1.77)	

Model 4: Model 3 + HDL-C; Model 5: Model 3 + LDL-C; Model 6: Model 3 + HDL-C + LDL-C.

### Subgroup analysis

**Figure 3:**
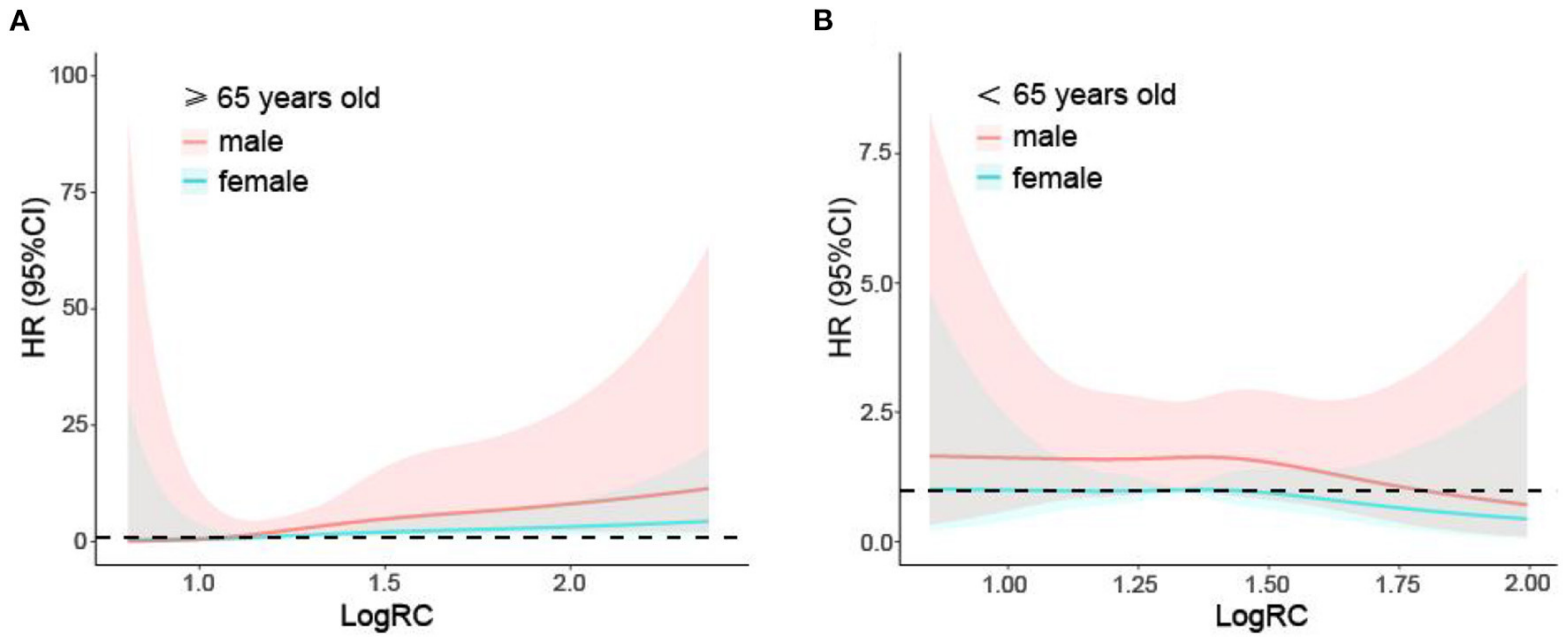
Restricted cubic spline plots of the association between log10-transformed RC and cardiovascular mortality in the general population over **(A)** and below 65 years **(B)** by sex. Analysis was adjusted for age + sex + race + antihypertensive medications + lipid-lowering medications use + systolic blood pressure + coronary heart disease + stroke + smoking. HR, hazard ratio; CI, confidence interval.

Analysis stratified by sex, age, diabetes, hypertension and smoking showed further evidence of a relationship between RC and cardiovascular mortality (Table 4). Males, participants over 65 years old, individuals with diabetes and hypertension, and smokers had a higher risk for cardiovascular death after full adjustment for several traditional cardiovascular risk factors (all *p* < 0.01). In addition, there were significant effects of the interactions of RC (*p* for interaction = 0.025) and log RC (*p* for interaction < 0.001) with age on cardiovascular mortality, and the population over 65 years old had a stronger association between log RC and cardiovascular mortality than that age 65 and younger. As shown by restricted cubic spline plots, there was a linear relationship between log RC and cardiovascular mortality in the subgroups stratified by sex, age, diabetes, hypertension and smoking (Figures 2B–F). This linear relationship was also found in population over 65 years old by sex (Figure 3A), but was not significant in those below 65 years old (Figure 3B).

**Table 4:** Subgroup analysis for cardiovascular mortality for logRC and RC (continuous variables).

	**LogRC**	***P***	**p-int**	**RC, mg/dL**	**RC, mmol/L**	***P***	**p-int**
	**HR (95% CI)**			**HR (95% CI)**	**HR (95% CI)**		
**Age**			<0.001				0.025
≥65 years	9.33 (3.22, 27.09)	<0.001		1.01 (1.01, 1.02)	1.49 (1.22, 1.82)	<0.001	
<65 years	0.57 (0.16, 1.95)	0.369		0.99 (0.97, 1.01)	0.67 (0.31, 1.44)	0.305	
**Sex**			0.080				0.158
Male	4.63 (1.68, 12.77)	0.003		1.01 (1.00, 1.02)	1.50 (1.19, 1.87)	0.001	
Female	0.96 (0.21, 4.49)	0.960		1.00 (0.98, 1.02)	0.93 (0.42, 2.07)	0.867	
**Diabetes**			0.086				0.112
Yes	5.36 (1.61, 17.82)	0.006		1.01 (1.00, 1.01)	1.44 (1.17, 1.77)	0.001	
No	1.27 (0.39, 4.15)	0.692		1.00 (0.98, 1.01)	0.88 (0.45, 1.71)	0.699	
**Hypertension**			0.803				0.705
Yes	3.08 (1.10, 8.62)	0.032		1.01 (1.00, 1.02)	1.45 (1.15, 1.82)	0.002	
No	3.39 (0.60, 19.24)	0.169		1.01 (0.99, 1.02)	1.24 (0.65, 2.36)	0.510	
**Smoking**			0.530				0.632
Yes	3.41 (1.19, 9.77)	0.022		1.01 (1.00, 1.02)	1.42 (1.12, 1.80)	0.004	
No	1.97 (0.47, 8.34)	0.356		1.01 (0.99, 1.02)	1.25 (0.74, 2.12)	0.408	

HR, hazard ratio; CI, confidence interval; p-int, p for interaction. Adjusted for age + sex + race + antihypertensive medications + lipid-lowering medications use + systolic blood pressure + coronary heart disease + stroke + smoking.

### Hazard ratios for cardiovascular events in different lipid groups

Corresponding to the LDL-C clinical cutoff point (130 mg/dL), the respective RC cutoff points were identified using equivalent percentiles and found to be 25.7 and 23.7 mg/dL in the male and female groups. The HDL-C clinical cutoff points were 40 and 50 mg/dL in the male and female groups. Compared with the reference group (lower LDL-C, lower RC and normal HDL-C levels), people with higher LDL-C, higher RC and abnormal HDL-C levels had a higher risk of cardiovascular mortality (HR 2.18; 95% CI 1.13–4.21 in men and HR 2.19; 95% CI 1.24–3.88 in women) (Table 5). In the case of normal HDL-C levels, the hazard of incident cardiovascular deaths of the male group with lower LDL-C and higher RC levels was also significantly different from that of the reference group (HR 2.34; 95% CI 1.29–4.24), as well as that of the female group with higher LDL-C and higher RC levels (HR 1.92; 95% CI 1.07–3.44). Notably, the female group with lower LDL-C, abnormal HDL-C and higher RC levels also had an increased hazard of cardiovascular mortality (HR 2.49; 95% CI 1.49–4.17). However, a larger sample size and more events in each category would improve precision.

**Table 5:** Hazard ratios (95% confidence interval) and *P*-value for cardiovascular events of different lipid groups.

	**Lipid group**		**Cardiovascular events, n /Individuals, n**		**HR (95% CI)**		***P*** **-value**	
LDL-C	HDL-C	RC	Male	Female	Male	Female	Male	Female
<130	≥ cutpoint	< cutpoint	73/3647	30/3,678	REF			
		≥ cutpoint	36/761	10/764	2.34 (1.29, 4.24)^**^	1.20 (0.59, 2.42)	0.005	0.618
≥ 130		< cutpoint	31/1552	21/1,327	1.25 (0.68, 2.30)	1.67 (0.97, 2.87)	0.469	0.065
		≥ cutpoint	25/765	18/800	1.47 (0.72, 3.02)	1.92 (1.07, 3.44)^*^	0.293	0.028
<130	< cutpoint	< cutpoint	15/795	8/1,325	0.82 (0.34, 1.95)	0.61 (0.28, 1.31)	0.648	0.202
		≥ cutpoint	32/1092	25/987	0.80 (0.38, 1.69)	2.49 (1.49, 4.17)^**^	0.554	0.001
≥ 130		< cutpoint	4/301	6/414	0.91 (0.28, 2.97)	1.40 (0.59, 3.33)	0.871	0.448
		≥ cutpoint	27/655	21/787	2.18 (1.13, 4.21)^*^	2.19 (1.24, 3.88)^**^	0.020	0.007

Corresponding to LDL-C clinical cutpoint (130 mg/dL), respective RC cutpoint was 25.7/23.7 mg/dL in male/female group. Respective HDL-C cutpoint was 40/50 mg/dL in male/female group. Adjusted for age + race + antihypertensive medications + lipid-lowering medications use + systolic blood pressure + coronary heart disease + stroke + smoking. ^*^P < 0.05, ^**^P < 0.01.

## Discussion

This is the first study using NHANES to reveal an independent association between RC and cardiovascular deaths in the general U.S. adult population, according to our results. In addition, RC showed a linear relationship with cardiovascular mortality that was more pronounced in males. Our results for patients with diabetes and hypertension, individuals over 65 years old, and smokers confirmed previous evidence on the association between RC and incident cardiovascular deaths, especially in people with higher LDL-C, higher RC and abnormal HDL-C levels.

After being remodeled in the systemic circulation, very low-density lipoproteins (VLDLs) undergo hydrolysis by the enzyme lipoprotein lipase (LPL), transforming into LDL, as well as intermediate-density lipoproteins (IDLs). RC represents the cholesterol content of the TRLs, which are made up of VLDL and IDL in the fasting state and chylomicron remnants in the non-fasting state. Overproduction of TRLs (24) (chylomicrons from the gut and VLDL from the liver) reduces the activity of lipolysis by LPL, which results in an accumulation of partially metabolized remnant particles. Particle for particle, these remnants exhibit an atherogenic potential similar to that of LDL particles (25), but they contain 40 times (26) more cholesterol. It can be suggested that the increased cardiovascular mortality risk associated with RC is caused by the mechanisms described before, which is related to local inflammation and plaque formation (27, 28). A higher concentration of RC in serum is more likely to penetrate into the arterial wall, where macrophages easily trap and absorb the RC, causing foam cells to form faster than they do with LDL (29). By hydrolyzing TRLs, RC induces the production of cytokines and interleukins, and proatherogenic adhesion molecules are released, activating inflammation and the coagulation cascade (30, 31). The combination of all these factors above may result in plaque rupture and ultimately, cardiovascular mortality.

Observational (3, 32) and genetic studies (27, 33, 34) have demonstrated an association between RC and cardiovascular outcomes. The fact that RC is causally associated with CVD and low-grade inflammation is now clear, regardless of fasting status, definition, and form of assessment (35, 36). Notably, however, the outcome in this study reflected cardiovascular mortality rather than just incident CVD, indicating that fatal rather than all cardiovascular events differed from those of previous studies. One interpretation for this is that our study included subjects with TG ≥400 mg/dL, and incident cardiovascular deaths in this population were reasonably counted. It would be more logical to estimate the RC value with the Sampson formula rather than Friedewald's equation because the LDL-C value computed by the latter is missing or inaccurate in this case (16). Another interpretation could be that most observational studies tended to miss a certain amount of cardiovascular deaths due to limited follow-up time or confined populations.

In previous cohort studies of obese elderly participants, RC ≥30 mg/dL distinguished individuals with a higher hazard of major adverse cardiovascular events (MACEs) than those with lower RC concentrations, irrespective of LDL-C levels (10). In this study, similar analyses were carried out. Those with higher RC and LDL-C and lower HDL-C concentrations faced the highest risk. This implied a complex interaction among RC, LDL-C and HDL-C. In addition to reversing cholesterol transport, HDL-C is also antioxidative and anti-inflammatory (37). However, recent epidemiological studies have demonstrated that the relationship between HDL-C and all-cause mortality and cardiovascular mortality is U-shaped in some specific populations (38–40). This could also partly explain why a similar situation occurred in our study when comparing the cardiovascular death risk of different lipid groups.

Various methods have been proposed for measuring and defining RC, including ultracentrifugation and immunoseparation (41). These methods, however, have been questioned for their accuracy, as remnant lipoproteins contain different apolipoprotein and lipid compositions. It has been proven difficult to measure RC directly because it takes a long time and requires special equipment. In 1990, NMRS (nuclear magnetic resonance spectroscopy, NMRS) was first used for the measuring of blood lipid components, and was subsequently standardized and used for the measuring of clinical blood lipid subgroup profiles. The principle is to obtain blood lipid profile levels by analyzing the specific amplitudes of different lipid methyl groups. NMRS can determine the number, density, size and composition of lipoproteins, and the results of NMRS are not easily affected by changes in lipoprotein composition (42). However, this method still has limitations. First, since NMRS is sensitive to drastic changes in the matrix, changes in ionic strength and pH can both affect the chemical shifts of lipid signals. Therefore, the detection should be completed within 4 h after blood collection (43). Secondly, if the laboratory does not have the conditions for testing, the plasma must be separated within the specified time and sent for inspection after cryopreservation. The freezing and thawing of the samples cannot be guaranteed during the storage and transportation of the plasma samples, and the accuracy of the test results may be affected (44), and finally, the NMR instrument is expensive, and it is still difficult to promote clinically. As a result, these methods cannot be used routinely (36). Comparatively, RC can be calculated as *TC*−*HDL*−*C*−*LDL*−*C*, as has been done in several large cohort studies (10, 11). Thus, accurately estimating LDL-C is crucial. The new Sampson formula reported by Sampson et al. is a better alternative for estimating LDL-C than conventional methods (18). Quite a few studies proved that Sampson's formula is more accurate than Friedewald's formula, even when TG is above 800 mg/dL (45, 46). Given the above reasons, it is reasonable to estimate RC using the Sampson formula. We found that RC significantly increased the risks of cardiovascular mortality, independent of both LDL-C and HDL-C, even after adjusting for lipid-lowering drugs and other confounders, and as a result, it would probably be more beneficial to use RC-targeted therapy than to further reduce LDL-C levels in high-risk subjects for whom statin treatment is not appropriate or who are already taking high- or moderate-dose statins (47–49). According to a recent study, lowering RC by 32 mg/dl can reduce recurrent MACEs by 20% in secondary prevention (50). There are certain therapeutic strategies available to lower RC levels by reducing TRL levels (12). Treatment with high-intensity statins limits triglyceride levels, whereas fibrates are more effective in lowering triglyceride levels and reducing cardiovascular hazards in people with atherogenic dyslipidemia (51–53). When statins and ezetimibe doses are optimized or intolerable, PCSK9 inhibitors may be used, but their triglyceride-lowering ability is modest (54, 55). On the other hand, high doses of omega-3 fatty acids, notably icosapent ethyl (54), and newer medications, such as RNA-based antisense-oligonucleotide inhibitors of apolipoprotein C-III and angiopoietin-like 3 genes (55, 56), markedly reduce TRL levels. Nonetheless, it must be determined whether this approach is superior to a more intensive LDL-C lowering strategy for CVD prevention, especially when risk-specific LDL-C targets are achieved in subjects with elevated TG levels at high CVD risk.

There are several limitations of our study. First, some variables were self-reported, which might cause recall bias. Second, despite adjusting for covariates, we cannot exclude the residual confounding effects from variables that were not measured or included, such as lipoprotein (a), apoA, apoB and apoE, due to limited data from the NHANES. Third, there may still be competing risks with other causes of mortality such as car accidents, although we have excluded cancer patients. Fourth, since the data were retrieved from a database of individuals from the U.S., they might not apply to other populations or regions. Fifth, this study was observational, and the predictive value of RC for cardiovascular mortality needs to be evaluated further in clinical studies. Finally, compared with direct measurement, our study might overestimate the value of RC due to the indirect method. Nevertheless, the indirect calculation of RC can provide valuable information for clinical management since it is affordable and inexpensive.

## Conclusions

We found that elevated RC levels increased cardiovascular mortality in the general population, independent of traditional cardiovascular risk factors, HDL-C and LDL-C. It may be more pragmatic to identify RC residual risk, as HDL-C boosting therapies have failed over the past few years and the era of targeted RC-lowering therapies is coming. A consensus on the most viable and cost-effective measurement method must be reached before RC is widely used in routine clinical practice. In the future, it will be necessary to demonstrate the mechanisms underlying the association between RC and cardiovascular mortality in addition to the total atherogenic particle concentration and to clarify the cardiovascular benefits of RC-targeted therapy.

## Data availability statement

The original contributions presented in the study are included in the article/supplementary material, further inquiries can be directed to the corresponding author/s.

## Ethics statement

The studies involving human participants were reviewed and approved by the Ethics Review Board of National Center for Health Statistics. The patients/participants provided their written informed consent to participate in this study.

## Author contributions

KZ contributed to formal analysis and writing-original draft of the manuscript. XQ and FZ were responsible for software and data curation. QD performed visualization. ZG, FW, and LX were responsible for methodology and investigation. ML contributed to validation. LC and YW contributed discussion and edited manuscript. HZ revised manuscript. YS and KZ contributed to conceptualization. XK designed and supervised the project. All authors contributed to the article and approved the submitted version.

## Funding

This paper was funded by the Science Foundation of Gusu School (GSKY20210105) and the Natural Science Foundation of Jiangsu Province (BK2012648). The funding body played no role in the design, writing, or decision to publish this paper.

## Conflict of interest

The authors declare that the research was conducted in the absence of any commercial or financial relationships that could be construed as a potential conflict of interest.

## Publisher's note

All claims expressed in this article are solely those of the authors and do not necessarily represent those of their affiliated organizations, or those of the publisher, the editors and the reviewers. Any product that may be evaluated in this article, or claim that may be made by its manufacturer, is not guaranteed or endorsed by the publisher.
